# Postprandial Oxidative Stress in Exercise Trained and Sedentary Cigarette Smokers

**DOI:** 10.3390/ijerph6020579

**Published:** 2009-02-06

**Authors:** Richard J. Bloomer, Kelsey H. Fisher-Wellman

**Affiliations:** Cardiorespiratory/Metabolic Laboratory, Department of Health and Sport Sciences, The University of Memphis, Memphis, TN, 38152, U.S.A.; E-mail: kfshrwll@memphis.edu

**Keywords:** Lipid peroxidation, oxidative stress, antioxidants, smoking, exercise

## Abstract

Cigarette smokers experience an exaggerated triglyceride (TAG) and oxidative stress response to high fat feeding. Exercise training may serve to attenuate the rise in these variables, by improving TAG clearance and antioxidant defense. We compared blood TAG, antioxidant capacity, and oxidative stress biomarkers in exercise trained (>2 hrs per wk) and untrained smokers matched for age, in response to a high fat test meal. We report here that low volume exercise training can attenuate postprandial lipid peroxidation, but has little impact on blood TAG and other markers of oxidative stress. Higher volumes of exercise may be needed to allow for clinically meaningful adaptations in postprandial lipemia and oxidative stress.

## 1. Introduction

Oxidative stress involves the production of reactive oxygen species (ROS) to an extent that overwhelms the antioxidant defense system [1]. Increased production of ROS promotes the oxidation of lipids and other molecules in ways that impair cellular function, possibly leading to disease [2, 3]. Cigarette smoking exacerbates ROS formation [4], evidenced by the increase in oxidative stress biomarkers in smokers compared with nonsmokers [5–7]. Cigarette smoke-induced oxidative stress poses a significant human health concern, especially as related to cardiovascular disease [8].

Aside from cigarette smoking, feeding with high fat meals induces oxidative stress (for a review please see reference [9]). This increase in oxidative stress may be mediated by the blood triglyceride (TAG) response [10, 11], as elevations in oxidative stress biomarkers seem to parallel those of TAG [12–14]. Individuals with elevated resting levels of oxidative stress (i.e., those with metabolic or cardiovascular disorders) have been shown to experience a greater response in oxidative stress biomarkers following intake of standardized meals when compared with healthy control subjects [15–17]. More specifically, cigarette smokers are known to experience impaired postprandial lipid metabolism [18, 19], mediated in part by a defective clearance of chylomicrons and their remnants [20]. Related to these observations, it has recently been reported that smokers experience an exaggerated TAG, as well as oxidative stress response to high fat feeding, compared with nonsmokers [21].

In an attempt to minimize oxidative stress post feeding, the logical solution is smoking cessation. This would likely improve lipid metabolism in response to feeding and decrease fasting levels of ROS [22, 23], as well as result in an improvement in the endogenous antioxidant defense system [24–26], allowing for a greater ability of the body to defend against ROS production. Despite these promising outcomes, the problem remains that smoking cessation rates are extremely poor [27]. Based on this reality, other methods to decrease smokers’ susceptibility to postprandial oxidative stress and related disease are needed.

One potential method to combat against postprandial oxidative stress in cigarette smokers is the performance of regular exercise. Although oxidative stress is increased acutely in response to strenuous exercise, this same stimulus appears necessary to allow for an up-regulation in endogenous antioxidant defenses [28–30]. In this way, the generation of ROS appears to be the “trigger” needed to allow for such adaptations, which may function to protect cells from future elevations in ROS. Hence, individuals who are exercise trained may exhibit attenuated oxidative stress following exposure to stressful conditions, a finding that has been repeatedly observed in nonsmokers [30].

Together with a potential for greater antioxidant capacity, exercise trained individuals process TAG more efficiently than untrained individuals following high fat meals [31]. This appears mediated in part by a reduced chylomicron-TAG half-life and by an increased activity of lipoprotein lipase, the rate limiting enzyme for serum TAG removal.

Considering the above, the purpose of this investigation was to compare blood TAG and oxidative stress biomarkers in exercise trained and untrained smokers of similar age, in response to a high fat test meal. We hypothesized that due to potential differences in endogenous antioxidant capacity and TAG processing, exercise trained smokers would experience an attenuated oxidative stress response following feeding compared with untrained smokers.

## 2. Methods

### 2.1. Participants

Exercise trained (n=10) and untrained (n=10) cigarette smokers participated in this cross-sectional study. Sample size was based on previous studies of postprandial oxidative stress in nonsmokers, as well as our prior work using smokers as subjects [21]. Unfortunately, due to the fact that no studies to date have investigated the effect of exercise training on postprandial oxidative stress in smokers, we had limited data to use in a power analysis for the current design. This is a limitation of the present study. Participants completed a health history and physical activity questionnaire and underwent a physical examination. Participants were normolipidemic (fasting triglycerides <150 mg·dL^−1^), nonobese, and did not have cardiovascular, metabolic, or pulmonary disease as defined by the American College of Sports Medicine [32]. No subject used nutritional supplements or medications (e.g., antiinflammatory or cardiovascular drugs). Participants needed to regularly smoke ≥ 5 cigarettes per day for a minimum of six months immediately preceding the test meal to be enrolled. Trained subjects needed to be currently performing a minimum of 2 hours of formal, structured exercise (not simply physical activity) per week for a minimum of 12 months. Although we initially set a higher criteria for exercise volume (≥3 hr per wk), the reality is that we simply could not identify interested smokers who were training at this higher volume. Therefore, some of the participants were exercising at a slightly lower volume. Following the screening procedures, participants were given detailed instructions and data forms related to the recording of dietary and physical activity data during the seven days before the test meal. All experimental procedures were performed in accordance with the Helsinki Declaration and approved by the University Human Subjects Review Board. Participants provided written consent prior to participating. Participant characteristics are presented in Table 1.

### 2.2. Test Meal

Participants reported to the laboratory in the morning (0700–0900 hours) following a 10-hour overnight fast. Subjects refrained from smoking for one hour prior to reporting to the laboratory. However, we did not record specific information regarding the exact time of the last cigarette (i.e., number of hours prior to reporting to the lab). Therefore, it is possible that some subjects may have smoked a cigarette on the morning of testing, prior to this one hour cessation period. This may be considered a limitation of the present study. Subjects were asked to abstain from strenuous exercise during the 24-hour period immediately preceding the test meal. Women reported during the early follicular phase of their menstrual cycle (days 1–5) in order to minimize any potential antioxidant effect of estrogen, as blood estrogen levels are lowest during this time. A resting pre meal blood sample was collected following a 10 minute quiet period. Participants then consumed the test meal (within 15 minutes). The meal consisted of a milkshake made with whole milk, ice cream, and whipping cream, as in our previous study with cigarette smokers [21]. The size of the milkshake was based on participants’ body mass, and equivalent to 1.2 grams of fat and carbohydrate, and 0.25 grams of protein per kilogram (amounts similar to quantities used in previously published studies). The shake provided approximately 17 kilocalories per kilogram. The observation period lasted six hours (from the start of meal intake), during which time blood samples were collected at 1, 2, 4, and 6 hours post meal. Participants remained in the laboratory during this period and relaxed. No additional meals or calorie containing beverages were allowed and smokers refrained from smoking cigarettes. Water was allowed ad libitum; with subjects consuming between 16 and 24 ounces.

### 2.3. Blood Sampling and Biochemistry

Blood samples (∼20 mL) were taken from an antecubital vein via needle and vacutainer pre-meal, and at 1, 2, 4, and 6 hours post meal. Blood was processed immediately and stored in multiple aliquots at −80°C until analyzed. Assays were performed in duplicate and on first thaw. Assays for TAG, glucose, total cholesterol, and HDL cholesterol were performed using serum following enzymatic procedures as described by the reagent provider (Thermo Electron Clinical Chemistry). Prior to HDL cholesterol analysis, precipitation of apolipoprotein B containing lipoproteins (VLDL, LDL, and Lp(a)) was performed using phosphotungstic acid, coupled with centrifugation, and HDL cholesterol was measured in the supernatant. LDL cholesterol was calculated using the Friedwald equation. Blood lipids were measured at rest (fasting) only, and used simply for group descriptive purposes. The exception was TAG, which was measured at rest (fasting) and at all times post feeding.

Superoxide dismutase activity (SOD) was measured in serum using enzymatic procedures as described by the reagent provider (Cayman Chemical, Ann Arbor, MI), where one unit of SOD is the amount of enzyme needed to exhibit 50% dismutation of the superoxide radical. Glutathione peroxidase activity (GPx) was measured in plasma using enzymatic procedures as described by the reagent provider (Cayman Chemical, Ann Arbor, MI). Values for GPx were calculated using the NADPH extinction coefficient and are presented in nmol·min^−1^·mL^−1^, where one unit is defined as the amount of enzyme needed to oxidize 1.0 nmol of NADPH to NADP^+^. Catalase activity (CAT) was measured in plasma using the Amplex Red reagent method as described by the reagent provider (Molecular Probes, Invitrogen Detection Technologies, Eugene, OR). All antioxidant enzymes were measured at rest (fasting) only, and used simply for group descriptive purposes.

Malondiadehyde (MDA) was measured in plasma following the method described by Jentzsch *et al*. [33]. Xanthine oxidase activity (XO) and hydrogen peroxide (H_2_O_2_) were both measured in plasma using the Amplex Red reagent method as described by the reagent provider (Molecular Probes, Invitrogen Detection Technologies, Eugene, OR). Antioxidant capacity was measured in serum using the Trolox-equivalent antioxidant capacity (TEAC) assay using procedures outlined by the reagent provider (Sigma Chemical, St. Louis, MO), and as previously described [34]. All oxidative stress variables were measured at rest (fasting) and at all times post feeding, as these are common biomarkers used in the study of postprandial oxidative stress.

### 2.4. Dietary and Physical Activity Records

Participants were instructed to maintain their normal diet and activity patterns, and to record these variables on log forms during the seven day period prior to the assigned test day. Nutritional records were analyzed for total dietary energy, macronutrients, and selected antioxidant micronutrients (Food Processor SQL, version 9.9, ESHA Research, Salem, OR). Activity logs were reviewed to determine the amount of activity done during the week prior to the test day. Subjects were given specific instructions to avoid physically stressful tasks during the 24 hour period preceding the test meal. It was important to control for any acute effects of physical activity on postprandial oxidative stress, as it has been recently reported that acute exercise may attenuate the rise in oxidative stress observed following feeding [13].

### 2.5. Statistical Analysis

Outcome variables were analyzed using a 2 (group) x 5 (time) repeated measures analysis of variance (ANOVA). Tukey’s post-hoc tests were performed when appropriate. Dietary, physical activity and descriptive data were analyzed using a one way ANOVA. The data are presented as mean ± standard error of the mean (SEM). Analyses were performed using JMP statistical software (version 4.0.3, SAS Institute, Cary, NC). Statistical significance was set at p≤0.05.

## 3. Results

With the exception of higher total (p=0.008) and LDL (p=0.012) cholesterol for untrained compared with trained smokers, no statistical differences were noted between groups for any descriptive variable (Table 1; p>0.05). In addition, no differences (p>0.05) were noted between trained and untrained smokers for dietary intake (Table 2; p>0.05). As expected, the amount of exercise performed by trained smokers was greater than for untrained smokers (Table 2; p<0.05), who reported no structured physical activity. The amount of self reported physical activity during the week prior to the test meal was similar to that of participants’ habitual exercise regimen.

**Figure 1.**
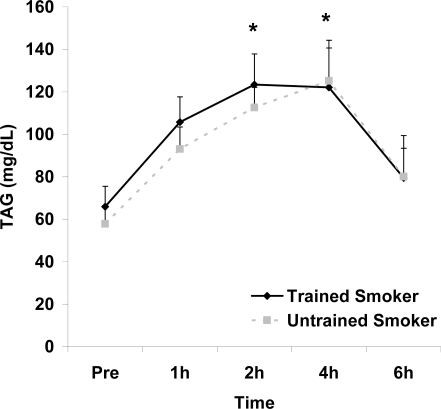
Serum triglycerides (mg·dL^−1^) before and following intake of a high fat meal in exercise trained and untrained cigarette smokers. Values are mean ± SEM. Time (p<0.0001) main effect; *Significant difference from Pre.

**Figure 2.**
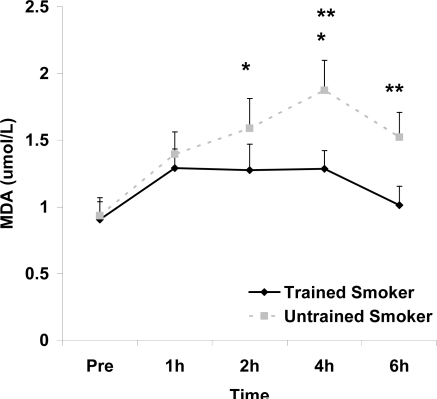
Plasma malondialdehyde (μmol·L^−1^) before and following intake of a high fat meal in exercise trained and untrained cigarette smokers. Values are mean ± SEM. Training status (p=0.0057) and time (p=0.0044) main effects; *Significant difference from Pre; **Significant difference between trained and untrained smokers at 4 (p=0.017) and 6 (p=0.039) hours post feeding using contrasts.

**Figure 3.**
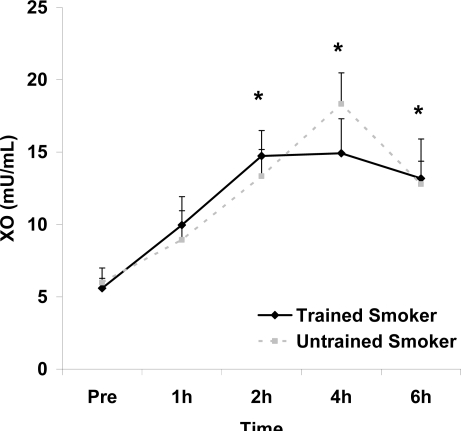
Plasma xanthine oxidase activity (mU·mL^−1^) before and following intake of a high fat meal in exercise trained and untrained cigarette smokers. Values are mean ± SEM. Time (p<0.0001) main effect; *Significant difference from Pre.

**Figure 4.**
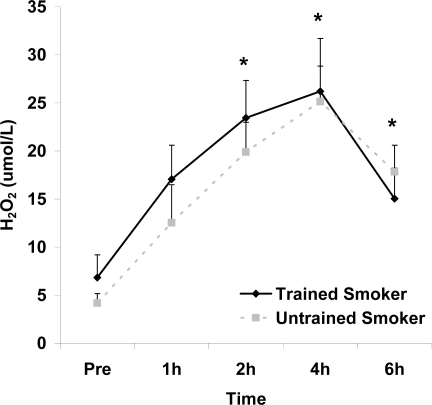
Plasma hydrogen peroxide (μmol·L^−1^) before and following intake of a high fat meal in exercise trained and untrained cigarette smokers. Values are mean ± SEM. Time (p<0.0001) main effect; *Significant difference from Pre.

**Figure 5.**
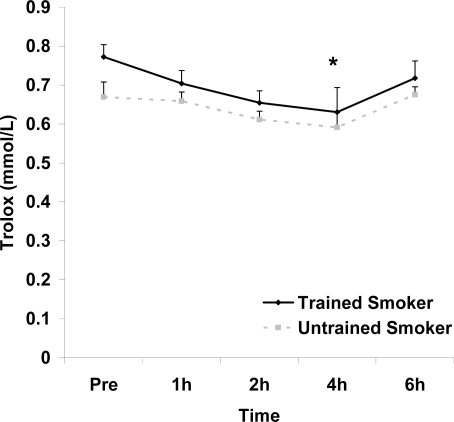
Serum Trolox^®^ equivalent antioxidant capacity (mmol·L^−1^) before and following intake of a high fat meal in exercise trained and untrained cigarette smokers. Values are mean ± SEM. Training status (p=0.0197) and time (p=0.019) main effects; *Significant difference from Pre.

**Figure 6.**
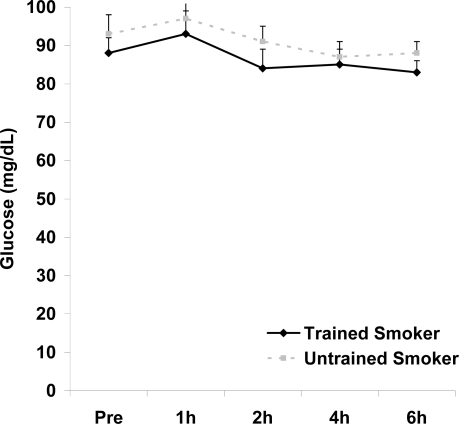
Serum glucose (mg·dL^−1^) before and following intake of a high fat meal in exercise trained and untrained cigarette smokers. Values are mean ± SEM. No significant differences noted (p>0.05).

Although antioxidant enzyme activities were slightly higher for trained compared with untrained smokers, no statistically detected differences were noted for CAT, SOD, or GPx activity (p>0.05; Table 3). No pre-meal differences were noted between groups for any outcome variable (p>0.05). Training status main effects were noted for MDA (p=0.0057) and TEAC (p=0.0197), with higher MDA and lower TEAC noted for untrained compared with trained smokers. Time main effects were noted for TAG (p<0.0001), MDA (p=0.0044), XO (p<0.0001), H_2_O_2_ (p<0.001), and TEAC (p=0.019). Post meal values were higher compared with pre meal at 2 and 4 hours for TAG (Figure 1) and MDA (Figure 2), and at 2, 4, and 6 hours for XO (Figure 3) and H_2_O_2_ (Figure 4; p<0.05). Values for TEAC were lower compared with pre meal at 4 hours post meal (Figure 5; p<0.05). Despite the above training status main effects, perhaps most importantly, there were no training status by time interactions noted for any variable. However, contrasts revealed group differences at 4 (p=0.017) and 6 (p=0.039) hours post meal for MDA. No effects were noted for glucose (p>0.05), and values were relatively unchanged over time for both trained and untrained smokers (Figure 6).

## 4. Discussion

Data from the present investigation indicate that exercise training at the volume performed by our subjects can influence postprandial lipid peroxidation, as measured by MDA. However, this volume is inadequate to influence postprandial TAG or other markers of oxidative stress in young cigarette smokers. This is the first study to our knowledge to report postprandial oxidative stress and TAG data in reference to exercise trained and untrained cigarette smokers. It is possible that a higher volume (or perhaps intensity) of exercise is necessary to impact postprandial TAG and oxidative stress in otherwise healthy cigarette smokers. This is an area of research that remains open for future investigation.

There are some potential explanations for our lack of group differences in selected biomarkers that deserve consideration. First, the difference in exercise training status between the two groups was demonstrated by an analysis of self-reported physical activity data. It is possible that trained subjects over-reported their activity levels and/or untrained subjects under-reported theirs. If so, marked differences in training status would not have been apparent. While including a measure of fitness such as VO_2max_ (the “gold standard” for aerobic capacity) would have been ideal, we simply did not have this information. We understand and admit that this is a limitation of the present study. Second, it is possible that our criteria for “trained” status (≥ 2 hours of structured, strenuous exercise per week) was too low, not allowing for optimal adaptations related to both TAG processing and antioxidant defense. However, our trained subjects reported an average of 2.5 and 1.1 hours of weekly aerobic and anaerobic exercise, respectively. Based on previous reports demonstrating effects for both TAG processing [31] and antioxidant enzyme up-regulation [30], it appears as though this volume of exercise would have been adequate to allow for positive adaptations, albeit not optimal. Although, it is uncertain as to whether or not subjects were exercising at the optimal intensity in order to promote such positive adaptations. Moreover, it is possible that although exercise training has been reported to positively influence TAG processing and antioxidant capacity in *nonsmokers*, exercise of similar intensity and duration may be ineffective for overcoming these known deficiencies in TAG processing and antioxidant capacity in smokers. Perhaps a greater volume and/or intensity of exercise is needed to allow for a comparable magnitude of change in these variables within a sample of smokers. Future work is needed to investigate this hypothesis. Subjects were asked to abstain from strenuous exercise during the 24-hour period immediately preceding the test meal to avoid alterations in postprandial TAG and oxidative stress values [35]. Although subjects indicated compliance to this recommendation, non-compliance to this important instruction could have influenced our findings. Finally, our relatively small sample size could have limited our ability to detect group differences in our chosen markers. This is indeed a limitation of this work, and should be considered relative to our findings.

Although a training status main effect was noted for TEAC, with trained smokers having higher values than untrained smokers, no statistically significant differences were noted between trained and untrained smokers for any of the antioxidant enzymes (Table 2). It is possible that greater differences in these enzymes between trained and untrained subjects are needed in order to observe a training status effect for these oxidative stress biomarkers. Related to the TAG processing, while it has been reported that regular endurance exercise training results in increased TAG utilization [31] this is mediated primarily by training volume [36]. Therefore, as suggested previously, the amount of exercise performed by our trained subjects may have been insufficient to demonstrate an effect for TAG. It is also important to note that all subjects in both groups were young and otherwise healthy, with low fasting TAG values (<150mg·dL^−1^), with no pre meal group differences noted. It is possible that significant differences between trained and untrained smokers would have been noted in the TAG response to feeding if greater disparity was apparent in fasting TAG values. It is also possible that findings may have been different if subjects were older, more established cigarette smokers. Future studies are needed to test these hypotheses.

In addition to a training status main effect for TEAC, the same was noted for MDA, with higher values for untrained compared with trained smokers. In addition, contrasts revealed group differences at both the 4 and 6 hour post meal time points for MDA. It is unknown what the potential importance of these isolated differences are.

## 5. Conclusions

In summary, we report that exercise training, at the volume performed by subjects in the present study, does not influence postprandial lipemia or oxidative stress in young cigarette smokers (as evidenced by the lack of interaction effects), with the exception of lipid peroxidation. This work may serve as pilot data for future studies. Such investigations should consider using a more controlled research design in which a higher number of untrained subjects are enrolled and randomized to an exercise training program, which is supervised, progressive, and of relatively high volume and intensity. This may allow for a more controlled method of investigating the effects of chronic exercise on postprandial lipemia and oxidative stress in cigarette smokers. Modification of the oxidative stress response to feeding may prove important for future morbidity and mortality.

## Figures and Tables

**Table 1.** Characteristics of exercise trained and untrained smokers.

Variable	Trained Smokers	Untrained Smokers
Age (yrs)	22±2	23±2
Height (cm)	173±3	175±4
Weight (kg)	74±5	75±7
BMI (kg·m^−2^)	25±1	24±1
Body fat (%)	19±2	17±2
Resting Heart Rate (bpm)	72±3	70±2
Resting SBP (mmHg)	112±3	114±4
Resting DBP (mmHg)	72±3	72±2
Total cholesterol (mg·dL^−1^)*	162±8	196±9
HDL cholesterol (mg·dL^−1^)	49±3	51±4
LDL cholesterol (mg·dL^−1^)*	100±8	133±11
Triglycerides (mg·dL^−1^)	65±8	58±9
Glucose (mg·dL^−1^)	88±4	94±6
Cigarettes per day	9±2	10±1
Years smoking	5±1	5±1

Values are mean ± SEM.

* No statistical differences were noted between groups for any above variable (p>0.05) with the exception of total cholesterol (p=0.008) and LDL cholesterol (p=0.012). Trained smokers were 4 men, 6 women; Untrained smokers were 6 men, 4 women. BMI, body mass index; SBP, systolic blood pressure; DBP, diastolic blood pressure; HDL, high density lipoprotein; LDL, low density lipoprotein. All bloodborne variables presented here represent fasting values.

**Table 2.** Dietary intake and physical activity of exercise trained and untrained smokers.

Variable	Trained Smokers	Untrained Smokers
Kilocalories	1990±239	1861±221
Protein (g)	85±12	74±13
Carbohydrate (g)	239±33	225±38
Fat (g)	64±7	67±11
Vitamin C (mg)	56±11	34±10
Vitamin E (mg)	4±2	4±1
Vitamin A (IU)	3882±1124	4794±1425
Selenium (μg)	39±11	40±12
Aerobic Exercise (yrs)	3.4±1.8*	0
Aerobic Exercise (h·wk^−1^)	2.5±0.6*	0
Anaerobic Exercise (yrs)	2.5±0.5*	0
Anaerobic Exercise (h·wk^−1^)	1.1±0.4*	0

Values are mean ± SEM. No statistical difference between groups was noted for any above dietary variable (p>0.05). All activity variables were greater for trained compared with untrained smokers (p<0.0001).

**Table 3.** Antioxidant enzyme activity of exercise trained and untrained smokers.

Variable	Trained Smokers	Untrained Smokers
Catalase (U·mL^−1^)	125.3±4.1	111.1±5.3
Superoxide Dismutase (U·mL^−1^)	0.134±0.009	0.120±0.011
Glutathione Peroxidase (nmol·min^−1^·mL^−1^)	139.3±9.9	134.7±11.1

Values are mean ± SEM. No statistical difference between groups for any of the above variables (p>0.05).
